# Forensic examination of plastic drinking straws based on their physical characteristics and manufacturing marks

**DOI:** 10.1080/20961790.2019.1595353

**Published:** 2019-05-29

**Authors:** Alaric C.W. Koh, Suat Ping Oh, Shing Min Lim, Sok Yee Yew, Jean Chen, Vicky Y.S. Chow

**Affiliations:** Forensic Chemistry and Physics Laboratory, Health Sciences Authority, Singapore

**Keywords:** Forensic sciences, drug packaging, plastics, manufacturing marks, tool marks, straw discrimination

## Abstract

This work highlights the evidential value of examining plastic drinking straws to establish linkages between multiple sources for forensic intelligence, investigative or prosecution purposes. Plastic drinking straws are commonly encountered in drug cases, either as inhalational paraphernalia or as packaging material. However, to the best of our knowledge, systematic studies on the evidential value of straws have not previously been carried out. In this study, over 80 packets of clear and colourless straws—most of which were visually similar—were purchased from various commercial outlets in Singapore. Some physical characteristics (viz., polarizing patterns, thickness, circumference and mass per unit length) and the manufacturing marks of these straws were examined to assess their potentials for discriminating straws from different packets. Comparison of polarizing patterns yielded a discrimination of approximately 69%, while thickness, circumference, and mass per unit length measurements resulted in lower discriminations. Comparison microscopy of manufacturing marks was found to be the most discriminating among all techniques employed herein, with a discrimination of about 95%, even among straws with similar polarizing patterns.

## Introduction

Drinking straws can be used as inhalational drug paraphernalia [1, 2], and also as packaging for drug trafficking purposes, both in Singapore [3] and other countries/regions [4–10]. Although the contents (i.e. the suspected illicit substances) of such straws would be analyzed, forensic examinations of the actual straw packaging *per se*, other than perhaps for DNA or fingerprints [6–8], have not been reported.

**Figure 1.**
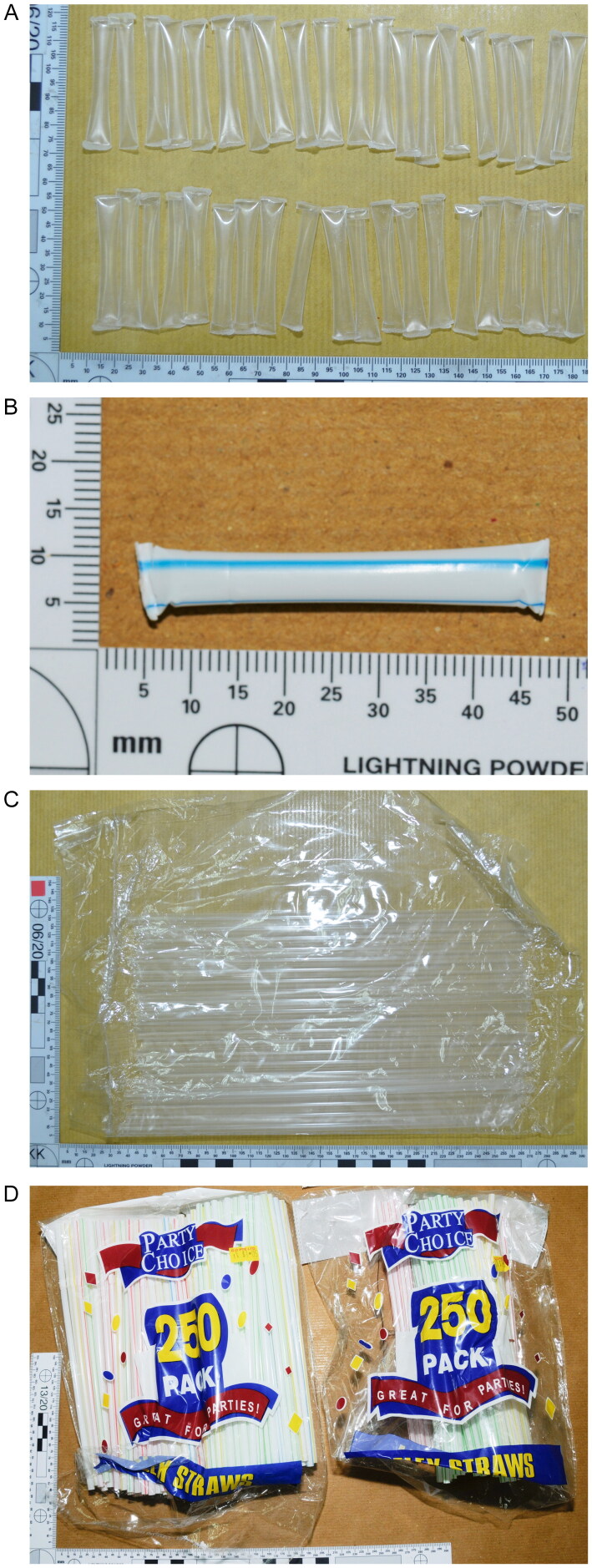
Case examples of (A and B) cut plastic tubes that had been heat-sealed at both ends, and (C and D) full-length drinking straws that were submitted for comparison.

The Forensic Chemistry and Physics Laboratory (FCPL) of the Health Sciences Authority, Singapore, received our first request for the examination of this evidence type from the Central Narcotics Bureau, Singapore, in 2009. The aim was to determine possible associations between a short straw segment containing drugs that was found on a suspected drug abuser, with 20 other straw segments that were recovered from a suspected drug trafficker. The request was made due to the absence of conclusive DNA and fingerprint evidence. FCPL’s examinations subsequently showed that these 21 straw segments were very likely to have originated from the same manufacturing source, and faced with this evidence, the suspected trafficker pleaded guilty. Since then, FCPL has received regular submissions of straw exhibits. These exhibits are typically in the form of cut plastic tubes (short straw segments) that had been heat-sealed at both cut edges using an open flame, examples of which are shown in Figure 1(A,B). In some cases, packets of full-length straws, such as those shown in Figure 1(C,D), were also submitted for comparison.

To the best of our knowledge, while the forensic examinations of other drug packaging materials—such as plastic bags [11–17], packaging films [17, 18], newspapers [19, 20], and adhesive tapes [21–27]—had been reported, corresponding studies of drinking straws have not been done. We thus compared the physical characteristics and manufacturing marks of drinking straws from various commercial sources available in Singapore to evaluate their discriminatory values, as well as to gain a better understanding of typical internal variations that might be encountered. The results of these studies are reported herein.

## Materials and methods

### Samples

A total of 83 packets of plastic drinking straws were purchased from 28 different commercial outlets located at different parts of Singapore. Each packet was assigned a unique identifier, and where necessary, individual straws were uniquely marked. In this work, the focus was on colourless and clear (transparent) straws, as these were largely indistinguishable by the naked eye.

These packets of straws, having been bought off-the-shelves, could be approximated as being representative of those that could be easily obtained by the general public. Indeed, we have had casework exhibits with similar packaging to some of these packets that were bought.

### Dimensions and mass

Length and mass measurements were obtained using rulers and an analytical balance (Mettler Toledo XP204, Columbus, OH, USA), respectively. To facilitate measurements of circumference and thickness, the straws were cut apart lengthwise. The circumference was approximated by measuring, with a ruler, the width of the cut straw. The thickness of the straw wall was measured using either a thin-film micrometer (Mitutoyo Corporation, Kawasaki, Japan) or a digimatic disk micrometer (Mitutoyo). For each packet, the above measurements were taken for either 50% or 20 straws (whichever was fewer), randomly selected from within the packet.

### Polarizing patterns

The polarizing patterns of 83 packets of straws were examined under a strain viewer (Sharples Stress Engineers Ltd., Preston, UK). All straws within each of the packets were examined to compare the polarizing patterns of straws (i) from the same packet, (ii) from different packets of the same commercial outlet, as well as (iii) from different commercial outlets.

### Examination of manufacturing marks

Manufacturing marks on the straws were examined using a comparison microscope (Leica FSC/Leica DM, Wetzlar, Germany). To facilitate comparison microscopy, the straws, which had been cut lengthwise for measurements, were sandwiched between two microscope glass slides.

### Data treatment

While a wide range of characteristics could, in principle, be used to compare two or more forensic samples, it would be unrealistic and impractical—in terms of manpower and resources (money, time, and/or equipment availability)—to do so. In order to objectively assess the potential of the different characteristics at maximizing long-term average discrimination, their “discriminating powers” (DPs) could be compared [28]:

$$\text{Discriminating power}=1-\;\frac{2M}{N(N-1)}$$

where *M* denotes the number of nondiscriminated pairs in the assessed characteristic and *N* is the total number of samples.

For characteristics such as thickness and circumference, the mean ± 3SD was calculated for each packet of straws. For the purpose of this paper, two packets of straws were considered to be nondiscriminated when their means ± 3SD overlap (Table 1).

**Table 1.** Example of two packets of straws which could be discriminated based on circumference measurements, but not using thickness measurements.

Identifier	Thickness (mm, mean ± 3SD)	Circumference (mm)	
		(Mean ± 3SD)	(Mean ± 4SD)
B8-1	0.121 ± 0.007	18.9 ± 0.9	18.9 ± 1.2
SKPJE-2	0.138 ± 0.021	21.5 ± 1.4	21.5 ± 1.8
B8-1 *vs.* SKPJE-2	Nondiscriminated	Discriminated	Nondiscriminated

Note the effect of using (mean ± 4SD) as compared to (mean ± 3SD). These two packets of straws could no longer be discriminated based on circumference measurements. In casework, to err on the side of discrimination (i.e. minimize Type I “false positive” errors), it might be preferable to use (mean ± 3SD) rather than (mean ± 4SD).

The DPs of polarizing patterns and manufacturing marks were calculated based on the results obtained independently by at least three qualified examiners. Any differences in opinion between the examiners were discussed and agreed upon, in consultation with other examiners.

Statistical significance for mass-based discriminations was determined using Student’s *t*-test (two unpaired populations of unequal variances). Results were considered significantly different at *P* ≤ 0.05. Pearson correlation coefficients were also used to infer possible correlations between characteristics. Calculations were performed using commercial software (Microsoft Excel 2007, Redmond, WA, USA).

## Results and discussion

### General observations

The number of straws found in each packet ranged from 42 to 183 (Table 2). The straws were usually packed in plain, clear and colourless, heat-sealed plastic bags. Commercial markings (brands “Penguin” and “Shuangyu”) were found on only nine of the 83 packets that were examined.

**Table 2.** The 83 packets of straws that were examined and some of their characteristics.

Packet’s identifier^a^	No. straws in each packet	Average circumference (mm)	Average thickness (mm)	Average mass per unit length (mg/mm)	Type of polarizing patterns
3STAR-1	120	21.0	0.124	2.07	1
3STAR-2	129	21.0	0.134	2.34	1, 9
3STAR-3	128	21.1	0.130	2.36	1
3STAR-4	129	21.0	0.131	2.33	1, 9
AM-1	137	21.4	0.127	2.30	2
AM-2	137	21.2	0.128	2.31	2
AM-3	138	21.4	0.125	2.28	2
ANB-1	50	13.0	0.068	0.70	1
ANB-2	50	13.0	0.067	0.71	1
ANB-3	52	13.0	0.065	0.70	1
ANB-4	50	13.0	0.069	0.73	1
ANB-5	52	13.0	0.067	0.71	1
B8-1	113	18.9	0.121	1.89	2
B8-2	132	21.0	0.133	2.31	2
B8-3	133	21.0	0.133	2.30	2
B8-4	144	21.0	0.135	2.28	2
EM-1	133	20.1	0.108	1.85	2, 11
EM-2	128	20.0	0.114	1.91	11
EM-3	165	19.4	0.115	1.85	8
EM-4	142	20.1	0.111	1.86	2
GK-1	123	19.1	0.132	2.06	5
GK-2	153	19.0	0.135	2.05	5
GK-3	154	19.1	0.133	2.06	5
HG-1	68	20.0	0.129	2.15	1
HG-2	61	20.1	0.129	2.17	1
KA-1	124	22.0	0.134	2.41	2
KA-2	131	21.9	0.118	2.14	2
KA-3	121	22.0	0.132	2.42	2
KHP-1	86	20.2	0.133	2.12	2
KHP-2	90	20.3	0.134	2.17	2
KHP-3	81	20.6	0.130	2.11	2
MSH-1	152	20.9	0.106	1.80	6
MSH-2	155	20.8	0.108	1.81	6
MSH-3	154	20.8	0.109	1.81	6
MWH-1	151	20.1	0.113	1.91	4
MWH-2	152	20.1	0.116	1.91	4
MWH-3	153	20.1	0.117	1.94	4
NSH-1	144	21.0	0.121	2.14	2
NSH-2	144	21.0	0.123	2.15	2
NSH-3	144	21.0	0.120	2.14	2
NTC-1	56	20.7	0.129	2.16	2
NTC-2	42	20.8	0.131	2.24	2
OJS-1	145	20.0	0.120	1.91	8
OJS-2	151	20.0	0.117	1.92	8
ONS-1	156	21.7	0.132	2.34	7
ONS-2	148	21.4	0.131	2.32	1
ONS-3	149	21.7	0.133	2.35	7
RS-1	141	21.9	0.127	2.35	1
RS-2	144	21.4	0.136	2.43	1
RS-3	145	21.4	0.136	2.44	1
SKPAMK-1	175	18.0	0.129	1.74	2, 8
SKPAMK-2	175	18.0	0.142	1.84	8, 10
SKPAMK-3	179	19.0	0.111	1.73	8
SKPB-1	173	19.0	0.110	1.74	2, 8
SKPB-2	148	20.5	0.137	2.28	8
SKPB-3	149	22.0	0.131	2.23	3
SKPBB-1	176	18.8	0.114	1.75	1
SKPBB-2	164	19.0	0.111	1.74	2
SKPBB-3	169	19.0	0.114	1.76	2, 8
SKPJE-1	135	21.0	0.127	2.24	2, 4, 8
SKPJE-2	141	21.5	0.138	2.29	2
SKPJE-3	182	19.0	0.114	1.74	2
SKPS-1	166	19.0	0.110	1.69	2, 8
SKPS-2	183	18.4	0.125	1.74	2, 8, 10
SSP-1	154	20.0	0.115	1.93	1
SSP-2	155	19.8	0.116	1.94	1
SSP-3	170	19.9	0.115	1.94	1
STP-1	149	20.0	0.121	1.97	1
STP-2	155	20.0	0.118	1.97	1
STP-3	144	20.0	0.118	1.97	1
UNI-1	138	22.0	0.129	2.27	2
UNI-2	138	21.9	0.130	2.27	2
UNI-3	136	21.6	0.128	2.25	2
VLD-1	169	20.0	0.138	2.27	1
WJ-1	153	19.0	0.112	1.77	1
WJ-2	148	19.0	0.115	1.84	1
WJ-3	149	19.2	0.114	1.83	1
YES-1	124	22.0	0.133	2.38	2
YES-2	125	21.9	0.133	2.39	2
YES-3	126	22.0	0.133	2.38	2
ZONE-1	136	22.0	0.149	2.61	1
ZONE-2	140	22.2	0.169	2.62	1
ZONE-3	145	21.2	0.135	2.42	1

a The identifier of each packet is assigned as ABC-N, where ABC is an abbreviated version of the commercial outlet it was purchased from, and *N* means that it was the *N*th packet purchased from that particular outlet.

### Thickness of straw walls

**Figure 2.**
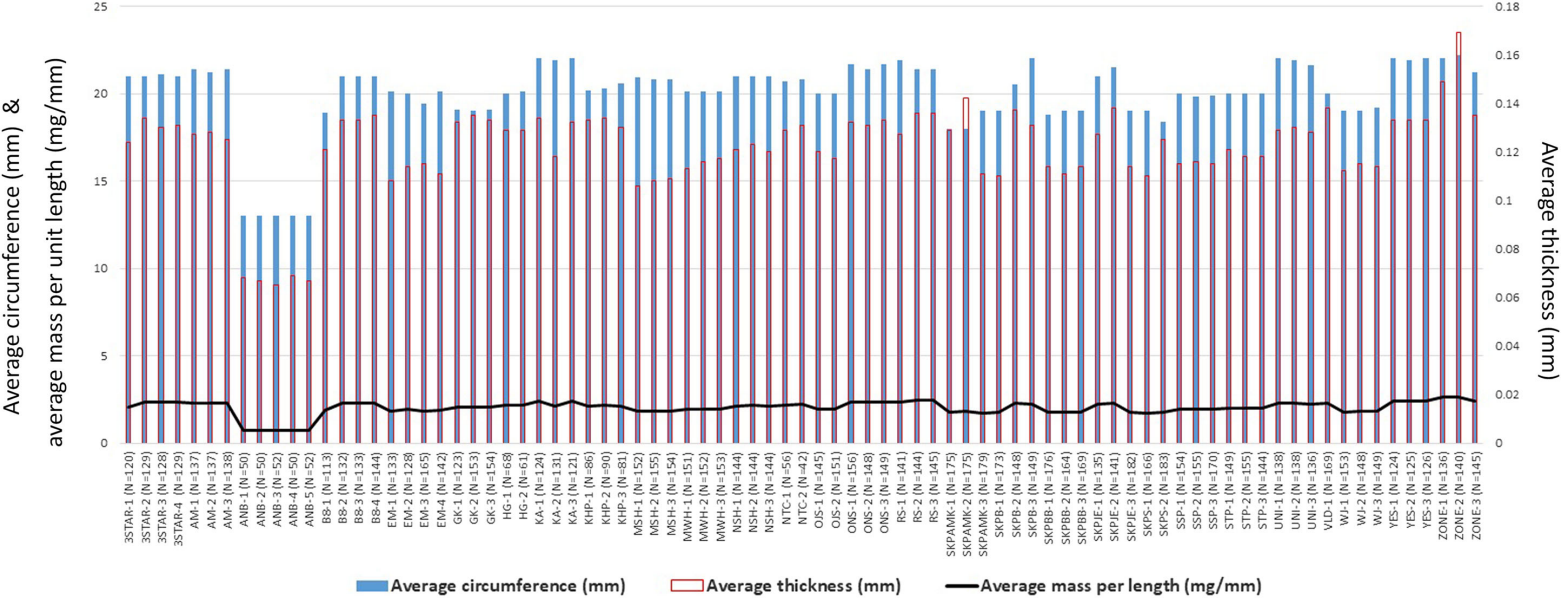
Average circumference, average thickness, and average mass per unit length of the purchased drinking straws.

The maximum measured difference in the thickness of the straw wall between straws from the same packet was found to be about 0.03 mm. As presented in Table 2 and Figure 2, the average thickness of the straws ranged from about 0.065–0.169 mm. The straws from all five packets bought from one particular outlet (“ANB”) had significantly thinner walls than the others, with average thickness of only about 0.065–0.069 mm. In comparison, there were 75 packets that fell within a relatively narrow range of about 0.106–0.138 mm. Overall, thickness measurement proved to be a poor discriminating technique, with a DP of 25%.

### Circumference of straws

The maximum difference in the circumference between straws from the same packet was found to be about 1 mm. As can be seen from Table 2 and Figure 2, straws purchased from the “ANB” outlet, with an average circumference of only about 13 mm, were significantly narrower than the others (about 18–22 mm). Overall, a DP of 58% was obtained, suggesting that it was a more discriminating characteristic than thickness measurements. Indeed, the circumference of straws in the market is likely to show greater variability: depending on the intended use, the circumference might range from about 5 mm (such as those for small bottled/packet drinks) to 36 mm or larger (such as those for “bubble tea”). As such, the afore-mentioned DP is likely to be an underestimate, since neither very narrow nor very wide straw types were considered in this report.

### Mass-based discriminations

**Figure 3.**
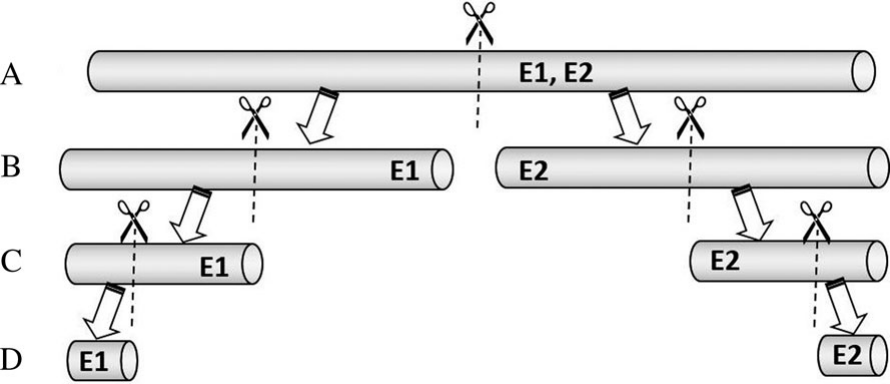
Schematic of the examination process to determine the effects of differing lengths of a straw segment on its mass per unit length. (A) The mass per unit length of a full-length straw was first determined independently by two examiners (represented by “E1” and “E2” in the above). The straw was then cut into two halves, from which the mass per unit length of the (B) half-length, (C) quarter-length, and (D) 1.5 cm long segments were consecutively determined.

Another easily measurable physical characteristic would be the mass of straws. As both full-length straws and cut straw segments are received in casework, mass per unit length comparisons were deemed more appropriate. The significance of the length of a straw segment on the resultant mass per unit length was evaluated. Twenty-two packets of straws from 16 different commercial outlets were used for this part of the study. One straw was taken randomly from each packet, for which the mass and length were measured for the full-length straw, a half-length segment, a quarter-length segment, as well as for a short segment of about 1.5 cm long, as illustrated in Figure 3. This last length was chosen as it corresponded approximately to length of the shortest straw that had been submitted to the laboratory for examination.

A DP of 60% was obtained based on measurements for the full-length straws. Although the DP appears relatively high, we noted in a separate experiment (data not shown herein) that the heat-sealing of the ends of straw segments using an open flame resulted in significant decrease in lengths (1 mm to 7 mm) due to melting, while changes to their masses were minimal. Caution should thus be exercised when directly comparing the mass per unit length of different straw exhibits, especially those of unsealed full-length straws with those of heat-sealed short straw segments. Should such comparisons be necessary, we would recommend that the measurements be made only after any heat-sealed ends of straw segments are cut off, and that full-length straws are cut to similar lengths.

### Polarizing patterns

**Figure 4.**
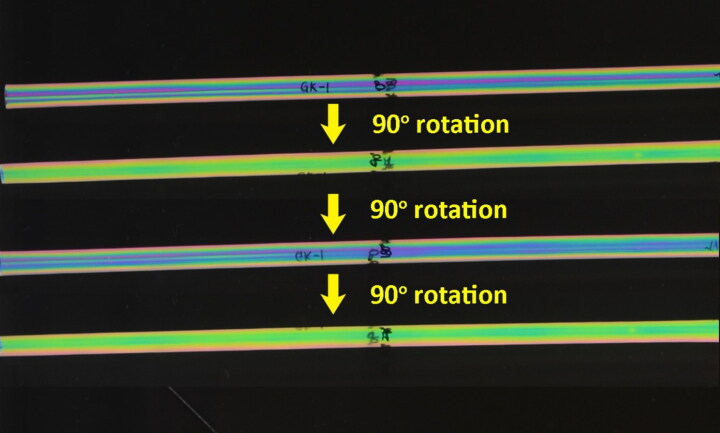
Variations in the polarizing pattern observed for a straw from the packet “GK-1” after a series of 90° rotations about its cylindrical axis.

**Figure 5.**
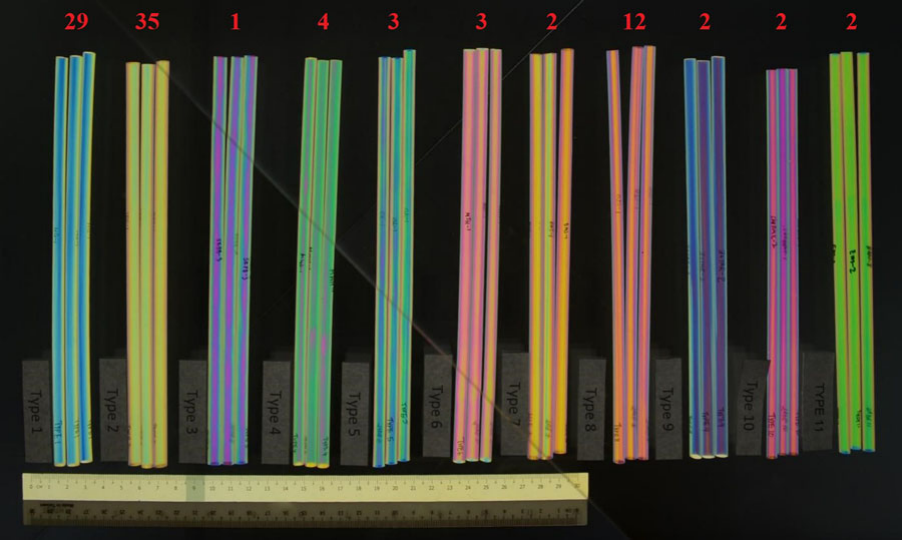
The 11 types of polarizing patterns that were observed (left to right: “Type 1” to “Type 11”). The numbers in red indicate the number of packets of straws for which the corresponding pattern had been observed. Note that the total adds up to more than 83, as two or more patterns were found in some packets.

All straws from each of the 83 packets were examined under polarized light and classified based on their polarizing patterns. As no sample preparation was required, this was found to be the most efficient method for comparing large quantities of visually-similar samples. We found that it was necessary to rotate each straw about its cylindrical axis during examination to fully account for internal variations (Figure 4). The straws were categorized into 11 different types of polarizing patterns, namely “Type 1” to “Type 11”, as shown in Figure 5.

#### Internal variations of the polarizing patterns of straws from the same packet

As shown in Table 1, about 88% of the packets (73 of the 83) contained straws with only one type of polarizing pattern, eight packets contained straws with two different polarizing patterns, while the remaining two packets contained straws with three different types of polarizing patterns. Overall, seven of the 14 packets from the “SKP” chain of stores (viz., “SKPAMK”, “SKPB”, “SKPBB”, “SKPJE”, and “SKPS”) contained straws with at least two types of polarizing patterns. This might be due to the sampling of straws for quality checks, the mixing of straws from two or more production lines, or the mixing of straws produced over an extended period of time prior to packing.

**Figure 6.**
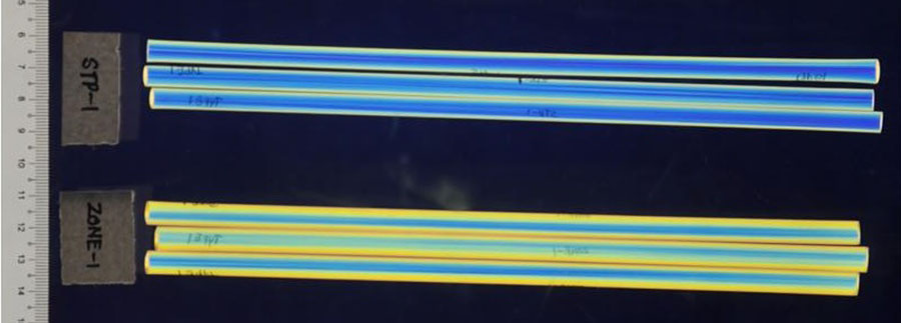
The straws from the packets “STP-1” and “ZONE-1” have observable differences in their polarizing patterns, and could, in fact, be discriminated. However, in this work, they are both classified as having “Type 1” polarizing patterns.

In order to estimate the DP, two packets of straws are considered to be discriminated only if the polarizing patterns of all the straws in the two packets are distinct. For example, “SKPB-1” contained straws with “Type 2” and “Type 8” polarizing patterns, and would not be discriminated with any other packets that had at least a straw of one of these types. Overall, the DP of this technique was found to be 69%, which is better than the other afore-mentioned methods. To avoid the possibility of incorrectly inflating of the DP, we have chosen to be more conservative—i.e. less discriminating—in our classification when calculating DPs (as pointed out by Mehltretter et al. [21], this approach is opposed to that taken in casework, which would be to err on the side of discrimination). In other words, certain packets of straws that were classified herein as having the same type of polarizing patterns could actually be discriminated based solely on polarizing patterns if they were compared pair-wise without considering the rest (Figure 6).

#### Polarizing patterns of straws with respect to the outlets they were bought from

In most cases, packets from the same outlet contained only straws of one type of polarizing pattern. For the outlet “ONS”, two types of patterns were observed for the three packets that were bought. At least three types of polarizing patterns were observed among straws bought from each of the “SKP” outlet.

#### Correlation of polarizing patterns with the other characteristics

The polarizing pattern of a birefringent material is known to change with its thickness, as reflected in the Michel-Levy chart. The thickness and circumference of a straw are, in turn, expected to affect its mass per unit length. These few characteristics were thus examined for possible correlations. As expected, thickness and circumference were found to be strongly correlated to mass per unit length, with Pearson Correlation coefficients of 0.95 and 0.93, respectively. A positive correlation between thickness and circumference (coefficient of 0.82) was also observed, possibly as wider straws should, in principle, require thicker walls to maintain the same rigidity. Correlations of polarizing patterns with these three other characteristics were, however, less evident. As can be seen from Table 1, straws classified as having the same type of polarizing patterns may have significantly different circumference, thickness and/or mass per unit length. For example, the mass per unit length of straws with “Type 1” polarizing patterns ranged from 0.70 mg/mm to 2.62 mg/mm. The lack of correlation might be due to differences in the polymer microstructures and/or varying states of internal stress.

### Comparison microscopy of manufacturing marks

As mentioned above, the examination of polarizing patterns was a simple and efficient method for discriminating straws from different commercial sources. There were, nevertheless, packets of straws that cannot be discriminated based on their polarizing patterns alone. As the examination of manufacturing marks is known to be useful when comparing plastic bags [11, 13, 14, 17], we hypothesized that comparison microscopy of manufacturing marks that were left on the straws during the extrusion process (notably, toolmarks left by the die, pullers, and sizing plates) will similarly be useful.

**Figure 7.**
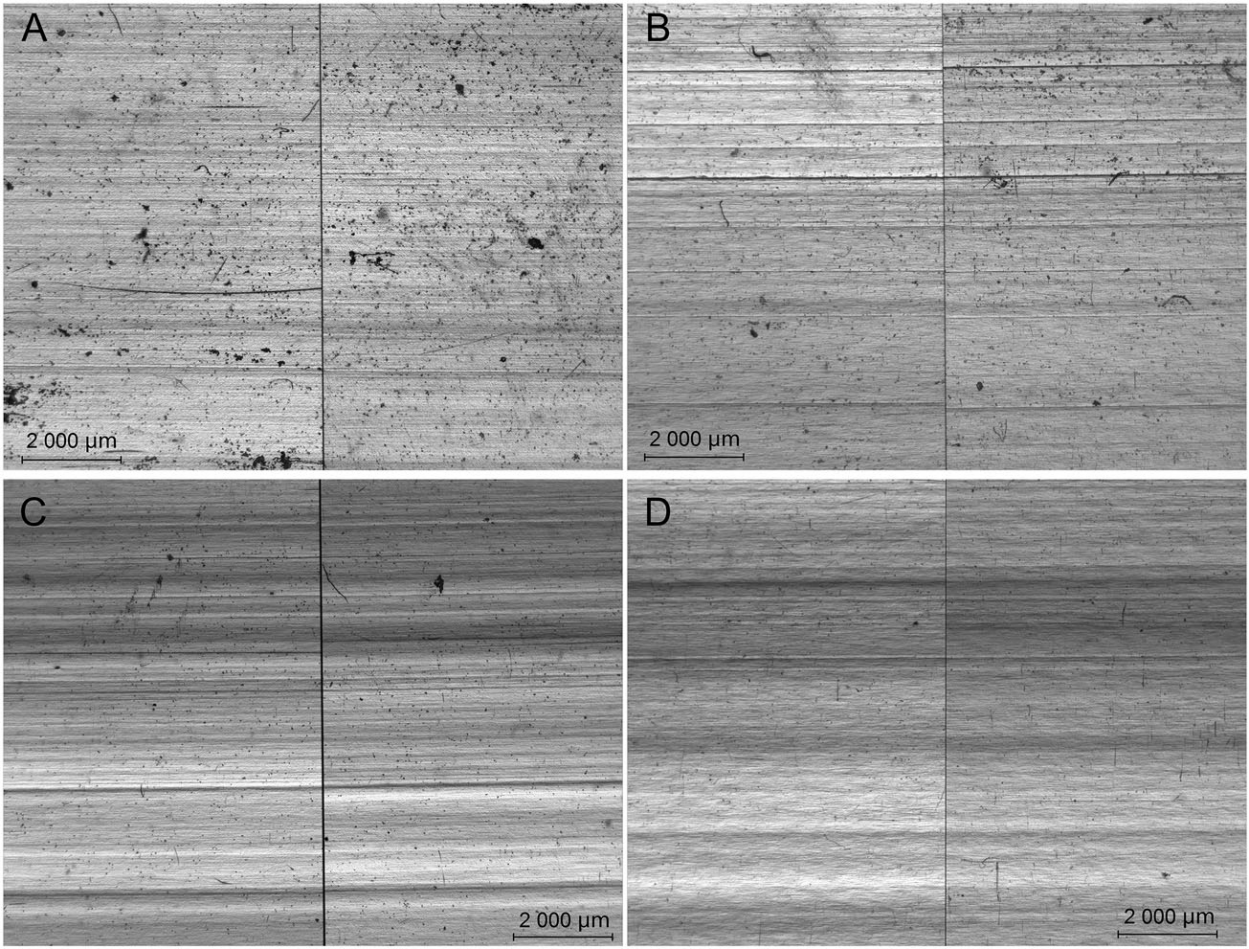
Photomicrographs of regions with similar manufacturing marks on representative pairs of straws from the packets labeled (A) “RS-1”, (B) “SKPBB-1”, (C) “SSP-1”, and (D) “WJ-1”.

**Figure 8.**
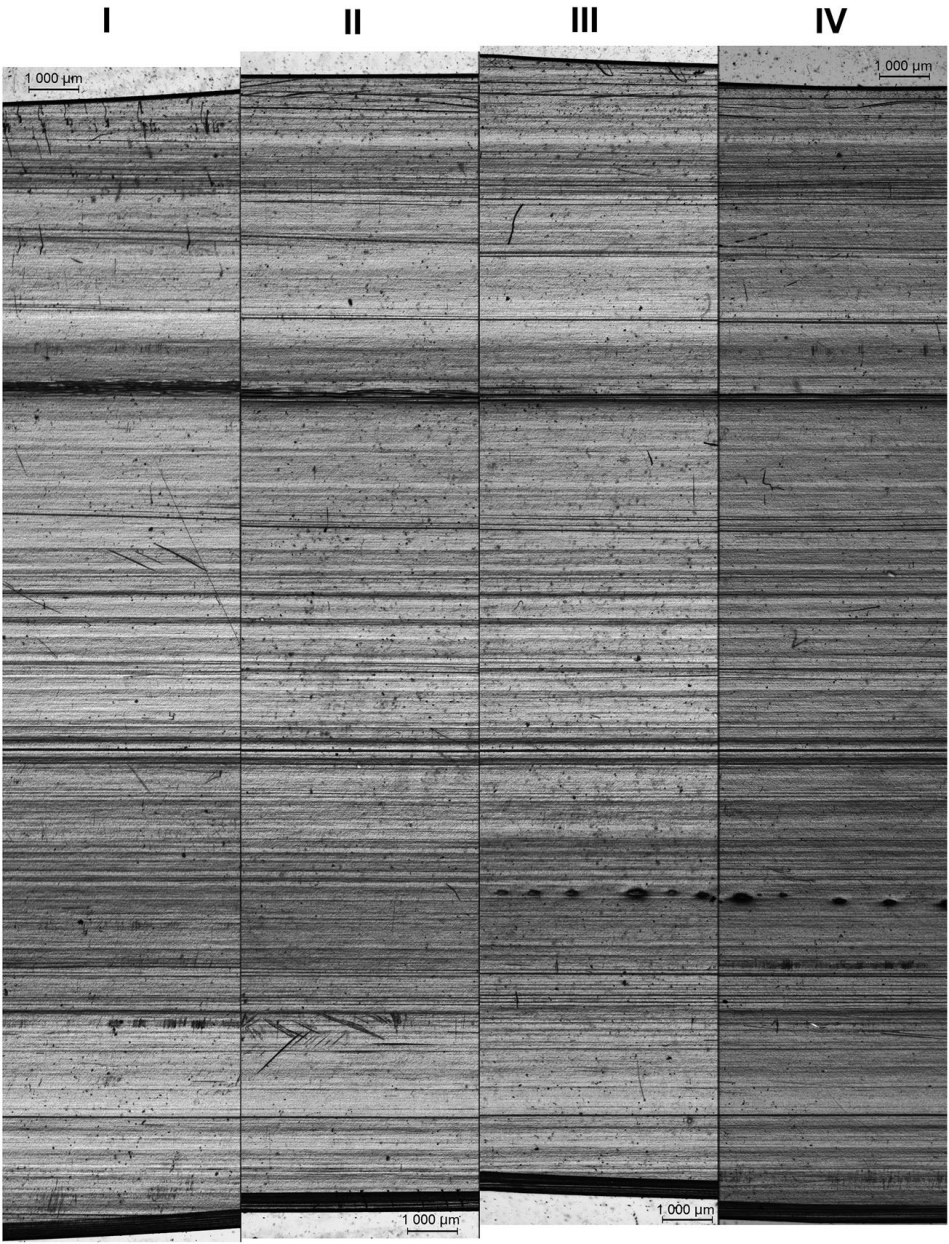
Stitched photomicrographs of the manufacturing marks at different regions of a single straw (which had been cut lengthwise) from the packet labeled “ZONE-1”. The distances between regions I to II, II to III, and III to IV were 3 cm, 2 cm, and 1 cm, respectively. Most of the striations were relatively straight and parallel to the extrusion direction, and were consequently well-aligned across the four regions. Some of the striations were, however, either wavy or produced at an angle to the extrusion direction; these were typically observed as being slightly displaced from a region to another. In general, better agreements were observed for regions that were nearer to each other.

For this study, 20 packets of straws from 10 different commercial outlets were chosen. These 20 packets consisted of straws with “Type 1” polarizing patterns, i.e. packets that could not be discriminated based on polarizing patterns alone. Fifty straws or 50% of the straws, whichever lesser, were randomly selected from each packet and compared. It was found that all straws from within each packet possessed similar manufacturing marks, four examples of which are shown in Figure 7. The presence of wavy and/or non-parallel manufacturing marks (Figure 8) resulted in slight shifts in the relative positions of some manufacturing marks between certain pairs of straws. We note that slight shifts might also be due to the fact that the straws were not completely flat despite being sandwiched between glass slides during examinations.

One straw was taken from each of these 20 packets and compared with one another. Of the 190 possible pairs, there were 10 pairs that possessed similar manufacturing marks and could not be discriminated using comparison microscopy. The 10 pairs of straws had originated from packets purchased from the same commercial outlet within a short period of time (Table 3). It is highly plausible that the packets of straws that could not be discriminated using this technique had in fact been manufactured by the same machine, within a period of time where the physical characteristics and manufacturing marks remained relatively similar.

**Table 3.** The 10 pairs of straws from the following packets could not be discriminated using comparison microscopy of manufacturing marks.

Packets	Remarks
“HG-1”, “HG-2”	Bought on the same day from the same commercial outlet.
“RS-2”, “RS-3”	Bought on the same day from the same commercial outlet. “RS-1”, which was also bought from this outlet on the same day, could be discriminated.
“SSP-1”, “SSP-2”	Bought on the same day from the same commercial outlet. Bought on the same day from the same commercial outlet. Bought on the same day from the same commercial outlet.
“SSP-1”, “SSP-3”	
“SSP-2”, “SSP-3”	
“STP-1”, “STP-2”	Bought on the same day from the same commercial outlet.
“WJ-1”, “WJ-2”	Bought on the same day from the same commercial outlet. Bought on the same day from the same commercial outlet. Bought on the same day from the same commercial outlet.
“WJ-1”, “WJ-3”	
“WJ-2”, “WJ-3”	
“ZONE-1”, “ZONE-2”	Bought 8 days apart from the same commercial outlet. “ZONE-3”, which was bought from this outlet on the same day as “ZONE-2”, could be discriminated.

Straws that had been purchased from different commercial outlets were all discriminated using this technique. Overall, this technique yielded a DP of 95% for straws that could not be discriminated based on their polarizing patterns. The use of comparison microscopy, a technique readily available to forensic laboratories involved in firearms/toolmarks work, is thus strongly recommended for the examination of straw exhibits.

It would be of interest to compare other analytical techniques that are applicable for the examination of polymeric materials [29], such as Fourier transform infrared spectroscopy (FTIR) and differential scanning calorimetry (DSC). These techniques are, however, beyond the scope of the current paper and will be the subject of a future work.

## Conclusion

Some physical characteristics (circumference, thickness, mass per unit length, polarizing patterns) and the manufacturing marks of clear and colourless plastic drinking straws were examined and compared. The two most useful parameters for discriminating visually similar straws were polarizing patterns (overall discrimination of 69%) and comparison microscopy of manufacturing marks (95% discrimination among straws with similar polarizing patterns). In summary, the results reported herein demonstrated that visually similar drinking straws may be associated or discriminated based on the comparisons of their physical characteristics and manufacturing marks. In Singapore, such examinations have been used to establish associations between drug traffickers and drug abusers. The examinations of straw exhibits should thus be considered for forensic, investigative or intelligence purposes, where appropriate.
